# Advanced glycation end-products accelerate amyloid deposits in adipocyte’s lipid droplets

**DOI:** 10.1038/s41419-024-07211-6

**Published:** 2024-11-19

**Authors:** Roza Izgilov, Nadav Kislev, Eman Omari, Dafna Benayahu

**Affiliations:** Department of Cell and Developmental Biology, Faculty of Medical and Health Sciences, Tel Aviv University, Tel Aviv, Israel

**Keywords:** Mechanisms of disease, Protein aggregation

## Abstract

Adipose tissue dysfunction is central to insulin resistance, and the emergence of type 2 diabetes (T2D) is associated with elevated levels of carbonyl metabolites from glucose metabolism. In this study, using methylglyoxal (MGO) and glycolaldehyde (GAD) carbonyl metabolites induced protein glycation, leading to misfolding and β-sheet formation and generation of advanced glycation end products (AGEs). The formed AGEs compromise adipocytes activity. Microscopic and spectroscopic assays were used to examine the impact of MGO and GAD on lipid droplet-associated proteins. The results provide information about how these conditions lead to the appearance of glycated and amyloidogenic proteins formation that hinders metabolism and autophagy in adipocytes. We measured the beneficial effects of metformin (MET), an anti-diabetic drug, on misfolded protein as assessed by thioflavin (ThT) spectroscopy and improved autophagy, determined by LC3 staining. In vitro findings were complemented by in vivo analysis of white adipose tissue (WAT), where lipid droplet-associated β-amyloid deposits were predominantly linked to adipose triglyceride lipase (ATGL), a lipid droplet protein. Bioinformatics, imaging, biochemical and MS/MS methods affirm ATGL’s glycation and its role in β-sheet secondary structure formation. Our results highlighted the pronounced presence of amyloidogenic proteins in adipocytes treated with carbonyl compounds, potentially reshaping our understanding of adipocyte altered activity in the context of T2D. This in-depth exploration offers novel perspectives on related pathophysiology and underscores the potential of adipocytes as pivotal therapeutic targets, bridging T2D, amyloidosis, protein glycation, and adipocyte malfunction.

## Introduction

Adipose tissue plays a central role in obesity pathophysiology and this tissue dysfunction promotes insulin resistance and type 2 diabetes (T2D), a chronic metabolic disease characterized by high levels of blood glucose (hyperglycemia) [1, 2]. Glucose metabolism generates a variety of carbonyl metabolites, including methylglyoxal (MGO) and glycolaldehyde (GAD). Methylglyoxal is also a byproduct of glycolysis which is impaired in T2D, results in its elevated levels [3, 4]. Chronic hyperglycemia accelerates this process and exposes the cells to high levels of carbonyl metabolites, which react with lipids and proteins to generate advanced glycation end products (AGEs) through the Maillard reaction [5–7]. Protein glycation causes alterations in protein structure and thereby promotes protein misfolding, β-amyloid formation and protein aggregation. Notably, such modifications occur in various proteins; for example, insulin and amyloid-β proteins, which have been associated with dysfunction due to protein alterations [8, 9].

Amyloidogenic proteins have the potential to create β-sheet structures, and the resulting protein assembles into insoluble fibers and aggregates that accumulate and induce cell toxicity [10, 11]. These cytotoxic amyloid aggregates are associated with a range of pathologies that are now recognized to extend beyond neurodegenerative diseases. Currently, around 50 proteins/peptides known to have the potential to deposit amyloid, including the T2D associated polypeptide amylin (IAPP) [12–14]. In this context, we recently demonstrated alterations in the protein secondary structure and generation of amyloids of serum albumin in presence of MGO and GAD [15].

The glycation byproducts promote protein structural modification and interfere with protein activity, leading to aberrations in the associated proteins interactions and signaling cascade [9, 16, 17]. Physiologically, such proteins should be recognized and eliminated by the autophagy system, which employs auto-phagosome formation and activation of the LC3 protein pathway. This highly regulated process, termed autophagy, is responsible for the degradation and recycling of cellular components and plays a critical role in maintaining cellular homeostasis. The process encompasses the clearance of misfolded or amyloid proteins, such as β-sheet aggregates, thereby preventing the accumulation of harmful entities that could promote pathologies such as in neurodegenerative disorders, Alzheimer’s and Parkinson’s diseases [18–22].

Chronic hyperglycemia in Type 2 Diabetes (T2D) has been recognized to lead to insulin insensitivity by damaging pancreatic β-cells [23–25]. In addition, an alteration in insulin signaling affects adipose tissue function leading to a vicious cycle of high glucose levels and consequently promotes hyper glycation and the formation of AGEs. The connection between AGEs formation and the deposition of amyloidogenic fibrils in adipocytes may be important in understanding the patho-physiology of adipose tissue. The glycation creates irreversible misfolded protein structures and the formation of aggregates as we recently have shown for serum albumin [15], and in an in vitro adipocytes’ cell system under glycated conditions [26].

Lipid droplets are essential adipocyte organelles that store and release lipids to meet the energy demands of the organism. The role of lipid droplets in adipose tissue metabolism is well-established, but recent studies have shown that lipid droplets are also associated with a variety of proteins that play critical roles in lipid metabolism, adipogenesis, and metabolic homeostasis [27–30]. The lipid droplet-associated proteins regulate various aspects of adipocyte biology, such as lipolysis, lipid uptake, and fatty acid oxidation, and are therefore critical for the regulation of adipose tissue metabolism. In addition, these proteins have been shown to interact with other cellular organelles, such as the endoplasmic reticulum and mitochondria, to coordinate lipid metabolism and energy homeostasis. Interestingly, alterations in lipid droplet function and size have been implicated in the development of adipocytes’ metabolic dysfunction. In a previous study we demonstrated that AGEs affect the level of adipogenesis [26], and here we use an advanced quantification approach to further examine the glycation effect on lipolysis and lipid droplet size [31].

Despite the strong association between T2D and amyloidosis, and although adipose tissue is a central player in insulin resistance and diabetes pathology, the formation of amyloidogenic proteins in adipose tissue has not yet been investigated. The current study was therefore designed to examine the effects of carbonyl compounds, glycation, and accumulation of amyloid structured proteins on the function of lipid droplets in adipocytes. The results provide new perspectives on amyloidogenic proteins and the conditions that promote such formation and affect the adipocytes’ function. We provide new information about the amyloid proteins that are associated with lipid droplets (LDs) and describe how hyper glycation impairs adipocyte function. We suggested that accumulation of misfolded proteins is a consequence of poor elimination by impaired autophagy as evidenced by MGO and GAD treated adipocytes and demonstrated the beneficial effect of the anti-diabetic drug metformin (MET) on this process. Among lipid droplet-associated proteins, we have identified ATGL as the primary protein that, when glycated, also tends to promote the formation of amyloid deposits in cultured adipocytes as well as in visceral adipose tissue.

## Results

**Fig. 1:**
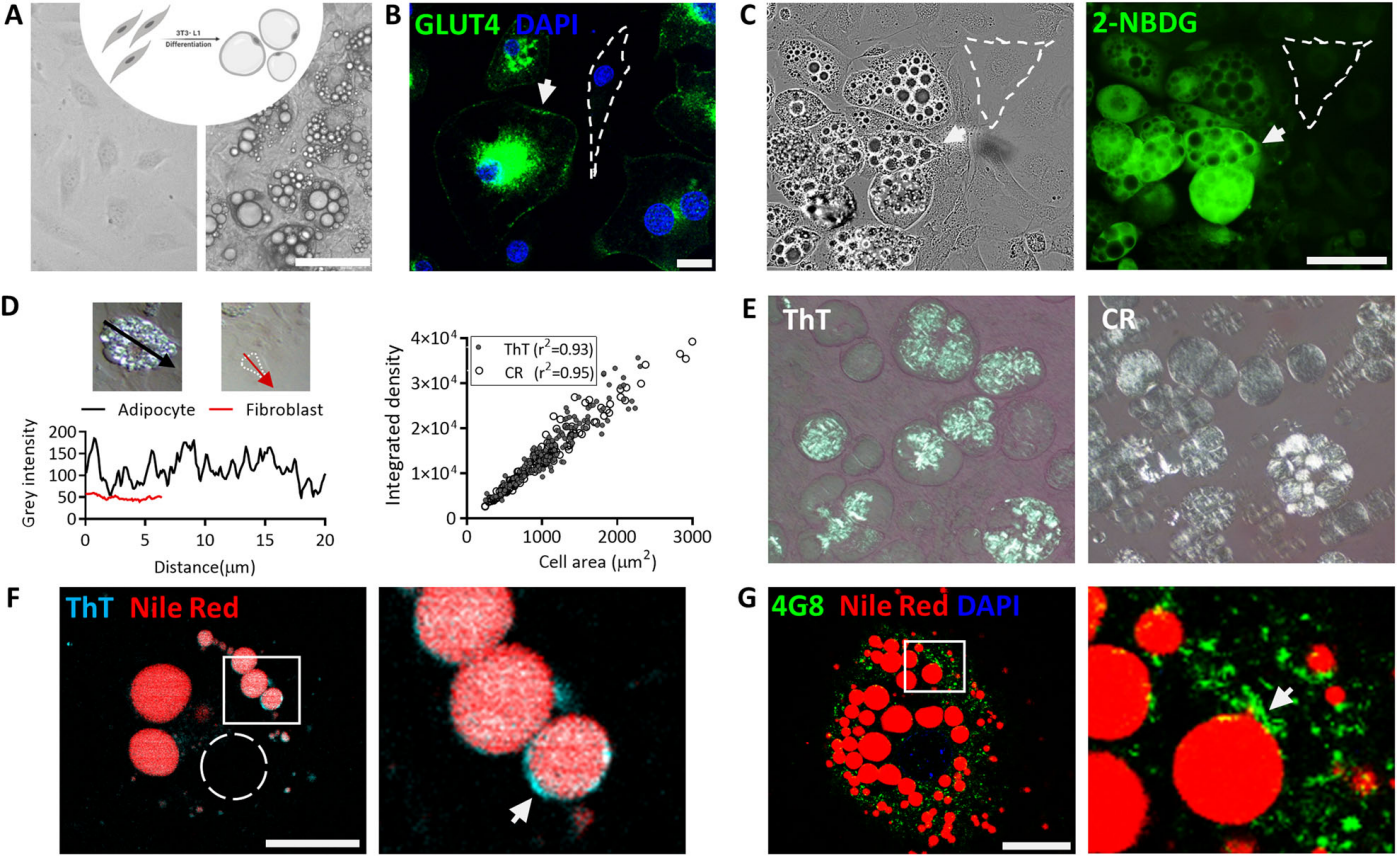
Adipocytes differentiation and amyloidogenic protein association with the lipid droplet. **A** Schematic illustration of 3T3-L1 cell differentiation, pre-adipocytes (left) to adipocytes (right) and culture images (Magnification of ×200, Scale bar = 125 µm). **B** GLUT4 expression shown by IF staining, dashed line marks a fibroblast, an arrow points on membrane GLUT4 staining in an adipocyte. Magnification of ×630. Scale bar = 20 µm. **C** Phase contrast images of adipocytes (left) and 2-NBDG uptake (FITC, right) differentiate between pre-adipocytes (dashed line) and adipocytes (arrow). Scale bar = 100 µm**. D**, **E** Polarized light microscopy images for Congo red (CR) and Thioflavin T (ThT) staining indicate amyloid formation (right panel). ThT staining intensity as measured in fibroblasts (red) and adipocytes (black), the staining is quantified as a function of cell area (Simple linear regression). left panel; magnification x200; ThT, *N* = 175; CR, *N* = 145 cells. **F**, **G** Confocal images of lipid droplet associated aggregates (arrows). Visualized by ThT probe (**F**) and 4G8 antibody IF (**G**). Lipid droplets stained by Nile-Red. Magnification of ×630. Scale bar = 20 µm. Dashed line marks the cell nucleus.

Pre-adipocytes have a flat elongated fibroblast-like shape (Fig. 1A left) that transitions morphologically to produce round mature adipocytes (Fig. 1A right) that contain lipid droplets (LDs). The accumulation of LDs is mediated by glucose uptake via the GLUT4 transporter, whose expression can be followed by assaying glucose uptake with 2-NDBG. The expression of GLUT4 is higher on the adipocyte plasma membrane and peri-nuclear storage as reflected by a stronger fluorescent signal than that seen on the fibroblast (Fig. 1B). Glucose uptake using the 2-NDBG followed by live-imaging shows a higher fluorescent signal in the adipocyte compared to fibroblast-like cells in the same culture (Fig. 1C, marked by a dashed line). The role of adipocytes in internalization of glucose motivated us to study the potential effect of hyperglycation due to the formation of AGEs.

We have previously reported that AGE formation increases the deposition of β rich amyloids and aggregates [15]. Here, we monitored the effects on adipogenesis in adipocyte cultured cells, specifically in 3T3-L1 cells treated with the carbonyl compounds MGO and GAD. Changes in protein conformation that reflect the development of amyloids can be monitored by staining adipocytes with Thioflavin (ThT) and Congo red (CR) and visualizing the resultant bright deposits with a polarized microscope (Fig. 1D, E). This is the gold standard for measuring β amyloid formation. Notably, the adipocytes exhibit bright staining while the fibroblasts remain dark. The brightness is associated with the adipocyte lipid droplets, although not all LDs exhibit the same level of staining. The basal level for fibroblast-like cells was 50 IU, while the adipocyte levels were four-fold higher, predominantly located in the membrane of lipid droplets, and with a positive correlation between cell area and integrated density (Fig. 1D). These results were verified by co-staining of Nile-Red (NR) with ThT (Fig. 1F) and by co-staining by IF with the 4G8 antibody (green), that has a structure specific binding to the β sheet alteration in amyloidogenic proteins (Fig. 1G) [32].

**Fig. 2:**
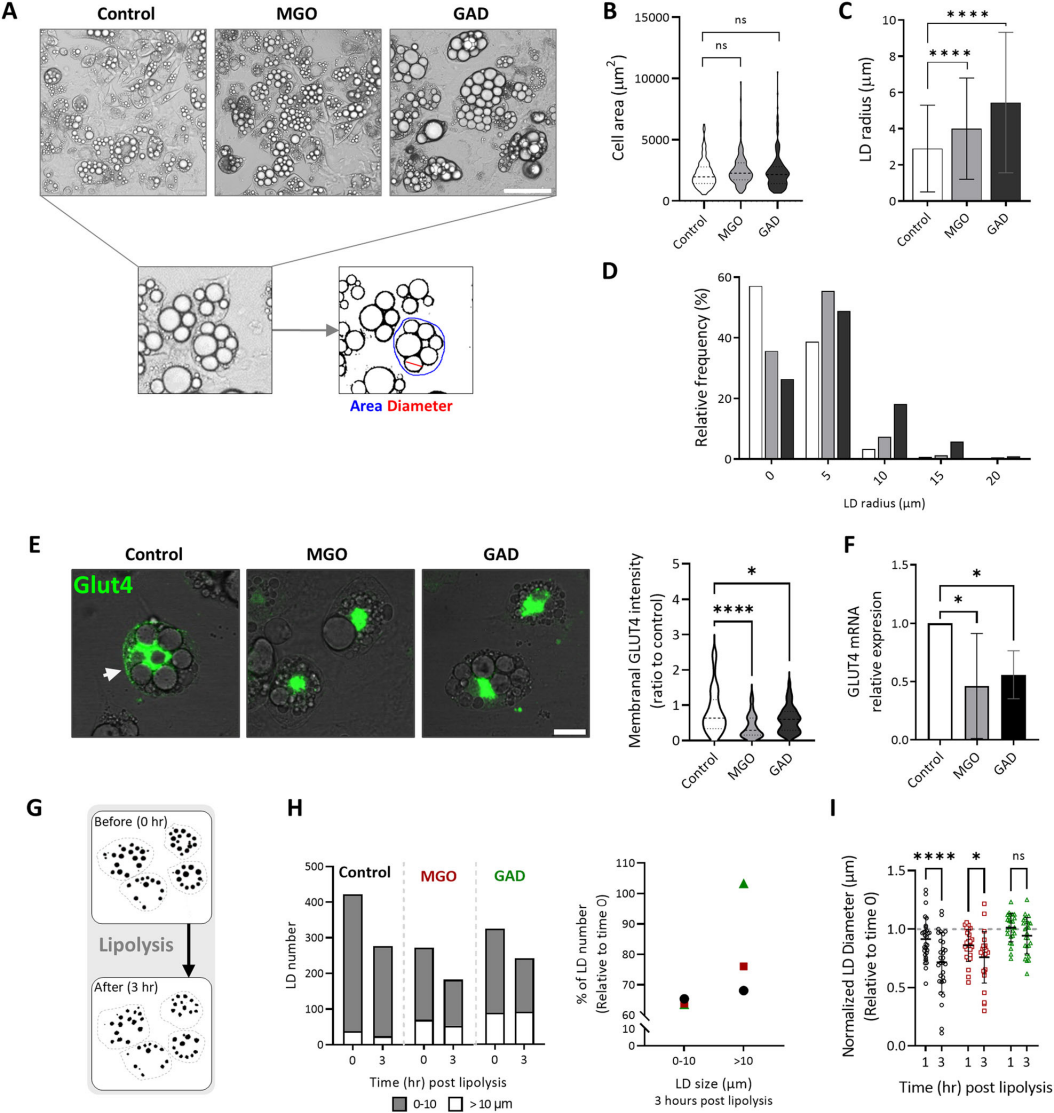
Carbonyl metabolites (MGO and GAD) influence adipocyte metabolism. **A** Phase contrast images of adipogenesis for control, MGO and GAD treated cultures. Magnification x200; Scale bar = 125 µm. **B** Measured cell area. **C** Mean LD radius and size distribution (**D**) of untreated and treated adipocytes. (Control-white; *N* = 114, MGO-gray; *N* = 155, GAD-black; *N* = 184 cells). **E** IF staining for GLUT4 membrane expression and quantification in MGO/GAD treated cultures (Magnification of ×630. Scale bar = 20 µm, *N* = 50 cells/group). **F** mRNA levels of GLUT4 in MGO and GAD treated adipocytes compared to controls (*N* = 4). (**B**–**F** One-way ANOVA). **G** Illustration of changes in LD content at the single cell level during lipolysis induction (dashed line- cell membrane). **H** Lipid droplet analysis during lipolysis measured by live imaging. Total LD number before and after 3 h of lipolysis induction in MGO and GAD treated adipocytes. The LDs are divided into two groups by diameter size (left panel; White - large LD, > 10 μm; Gray - small LD, 0 < 10 μm). LD number change (% from the starting point) after 3 h of lipolysis induction in small and large LDs (right panel; - control; - MGO; - GAD). **I**. LD diameter after 1 and 3 h of lipolysis induction relative to the starting point. Two-way ANOVA. Data are presented as mean ± SD. (ns) *p* > 0.05; *p < 0.05; ****p ≤ 0.0001.

The involvement of adipocytes in the internalization and metabolism of glucose led us to examine how the formation of AGEs can affect cellular activity and specifically the differentiation capacity of 3T3-L1 cells. As already discussed, we have previously reported that treating cell cultures with the carbonyl compounds MGO and GAD decreases the level of adipogenesis (LOA) [26]. Here, we extended the study to changes in adipocyte morphology and specifically to effects on cellular area and LDs size. Single-cell analysis enables us to characterize the changes in LDs accumulation and cell morphology (Fig. 2), did not detect any differences in cellular area. In contrast, incubation with MGO or GAD increased the LDs radius by 40%, with values of 4 µm ± 0.06 or 5.4 µm ± 0.09 after exposure to MGO or GAD respectively, compared to a mean radius of 2.9 µm ± 0.05 in untreated adipocytes (Fig. 2A–C). The frequency distribution of LD size was also increased after treatment (Fig. 2D).

The GLUT4 transporter depends on insulin for glucose internalization into adipocytes, and transporter translocation to the cell membrane from cytoplasmic storage vesicles is a crucial step in the process. Figure 2E presents the IF staining for GLUT4 and quantifying the expression on cell membrane, indicated that treatment with MGO and GAD reduces the GLUT4 expression on the adipocyte plasma membrane by a factor of two compared to control cells. This was accompanied by a decrease in GLUT4 mRNA level in the treated cells (Fig. 2F). A reduction in GLUT4 transcription and cell membrane expression reflects the development of insulin signaling impairment. In addition to glucose uptake, an alteration in cell signaling also affects adipocyte lipid metabolism and storage by lipolysis. This was investigated by inducing the lipolysis process in treated adipocytes and measuring the morphological changes over 3 h by single LDs analysis in live imaging (Fig. 2G–I). If the LDs are categorized as small or large by radius size (0–10 and >10 µm), the smaller LDs proved more sensitive to lipolysis induction than the large LDs regardless of treatment. However, 8% fewer large LDs reacted to lipolysis in MGO treated adipocytes, while GAD had no effect on the number of large LDs compared to control (30% less, Fig. 2H). This profile is obvious from the change in LD diameter presented in Fig. 2I, with a significant reduction in control cells compared to a moderate change in MGO treated cultures and no change in the cells treated with GAD. The proteins responsible for lipolysis are the LD-associated proteins that interact with the LD membrane, and this fact combined with the amyloid formation in Fig. 1, led us to look for changes in amyloid formation in adipocytes treated with carbonyl compounds.

**Fig. 3:**
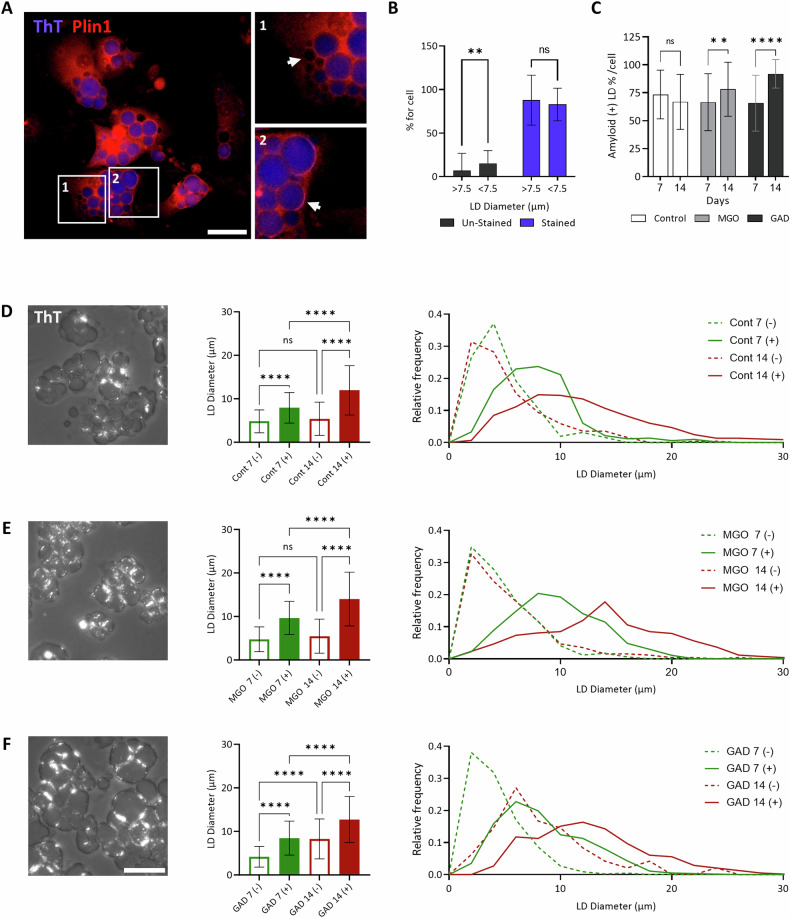
Amyloid-positive appearance on lipid droplets following exposure to carbonyl metabolites. **A** Adipocytes stained for perilipin 1 (plin-1) and ThT: 1. Unstained LD (marked by an arrow); 2. Stained LDs with co-localization of ThT and IF for plin-1 in the LD membrane (marked by an arrow, magnification of ×200. Scale bar = 50 µm). **B** Quantification of the percentage of ThT+ stained and unstained LDs (Diameter: large LD, >7.5 μm; small LD, <7.5 μm; *N* = 156 cells). **C** Quantification of LD with ThT+ staining of amyloid (polarization images in **D**–**F**) after 7 and 14 days with MGO or GAD. Data are quantified as % of total LD count per-cell. Two-way ANOVA with Tukey’s post hoc test. **D**–**F** Polarized images of ThT stained adipocytes after 14 days treatment (left), LD diameter quantification (middle panel) and distribution (right). The quantification of ThT+ and ThT- after 7 (green) and 14 (red) days of treatment - control (**D**); MGO (**E**); GAD (**F**). Scale bar = 50 µm. One-way ANOVA. Data are presented as mean ± SD. (ns) *p* > 0.05; **p ≤ 0.01; ****p ≤ 0.0001.

The lipid droplets were co-stained with ThT and with anti-plin-1, a lipid droplet membrane protein. ThT staining is related to LD size and is at higher level on the large LDs, although not all LDs are stained with ThT (Fig. 3A, B). The heterogeneity in staining may be attributed to the level of amyloid formation during LDs maturation. Further, the effect of carbonyl metabolites on LDs were assessed on differentiated adipocytes at two time points: 7 and 14 days of treatment (Fig. 3C–F), and we analyzed LDs diameter correlated with the presence of amyloid aggregation. Amyloids formation was detected as ThT^+^ by a polarized microscope. No changes in the level of amyloid positive LDs were detected in control cells over the course of the experiment. In contrast, treatment with MGO or GAD increased the levels of ThT^+^ LDs at both time points: MGO (from 66.6% to 78.2%) and GAD (from 65.7% to 92.0%, Fig. 3C). Images of ThT^+^ stained LDs at day 14 (Fig. 3D–F), which presents the difference in ThT staining and the amyloid accumulation after treatment with MGO and GAD.

Similarly, the distribution of LDs diameter at the two time points (Fig. 3D–F) detected no difference in the distribution of ThT^-^ LDs in control cells over the course of the experiment, although the diameter of the ThT^+^ LDs increased after 14 days (from 7.9 µm to 11.9 µm). MGO treatment caused a significant shift only in the ThT^+^ LDs after 14 days (from 9.7 µm to 14.0 µm), and it became clear that the ThT^-^ LDs in both the control and MGO treated samples represent the smaller LDs (peak around 4–7 µm). In contrast, GAD treatment resulted in a size shift in both ThT^+^ (from 8.5 µm to 12.7 µm) and ThT^-^ (from 4.2 µm to 8.3 µm) LDs with time. These results support the LDs radius differences, and lipolysis results described above (Fig. 2) and demonstrate a variation in the LD-amyloid aggregation caused by MGO or GAD.

**Fig. 4:**
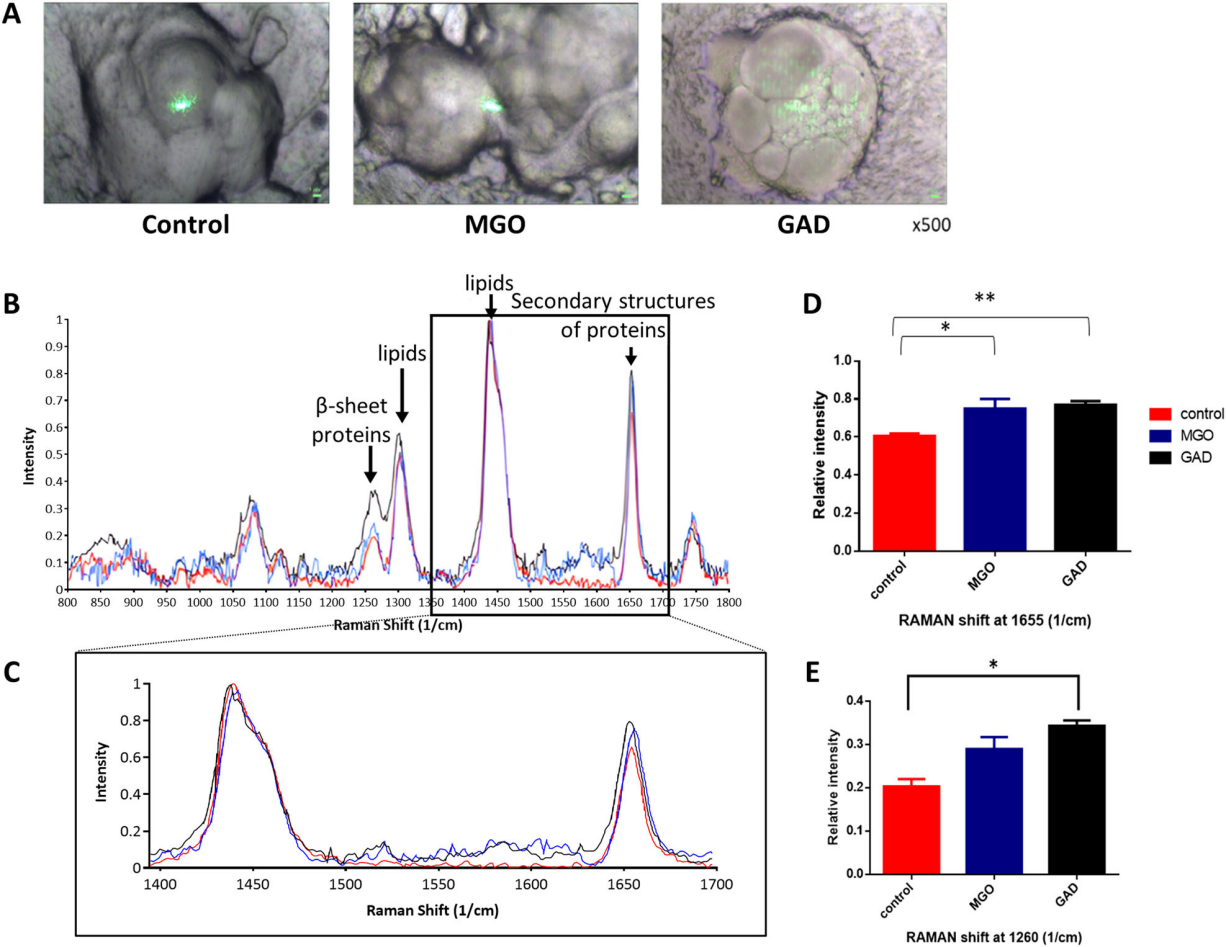
RAMAN spectra of carbonyl metabolites treated live adipocytes. **A** RAMAN microscopy bright-field images of untreated and MGO/GAD treated adipocytes (Magnification of ×500). **B** RAMAN spectral profile comparison of untreated and treated adipocytes measured with a 532 nm excitation laser. Structure specific peak positions are labeled. **C** Magnification of the spectral range 1400–1700 1/cm. **D** Normalized intensity of RAMAN band at main peak at 1655 1/cm represents the secondary structures and the peak at 1260 1/cm (**E**) represents the β-sheet structure (*N* = 5–10 scanned areas). Control (red); MGO (blue); GAD (black). Data are presented as mean ± SD. One-way ANOVA, *p < 0.05; **p ≤ 0.01.

The ThT^+^ staining between the treated groups indicates the β-sheet/amyloid accumulation associated with the LDs were further verified by RAMAN spectral analysis. Figure 4A shows the images taken on RAMAN microscopy of untreated and MGO/GAD treated adipocytes. The Raman spectral analysis allows us to analyze the structural and chemical “fingerprint” of the proteins and detect any alterations in chemical composition and molecular properties. Intracellular changes in the secondary structure of proteins caused by MGO/GAD treatment are reflected by the molecular profile in the measured spectrum. The results revealed that untreated or treated adipocytes have a similar peak at 1440 cm^−1^ which reached 0.99, indicating that all groups have the same lipid content (Fig. 4B). However, significant differences in the chemical bonding and intramolecular bonds could be observed by analyzing the amide I peak at 1655 cm^−1^, which is associated with the secondary structures of proteins. The peak intensity was 1.3-fold higher in adipocytes treated with MGO or GAD than in untreated controls (Fig. 4D). Similarly, the RAMAN signature at 1260 cm^−1^, which represents amide III and intramolecular β-sheet structures (Fig. 4C), was 1.8-fold stronger in adipocytes treated with GAD compared to untreated adipocytes (Fig. 4E). The results represent the differences in the secondary structures present in treated adipocytes.

### Carbonyl compounds influence the intracellular degradation pathway

**Fig. 5:**
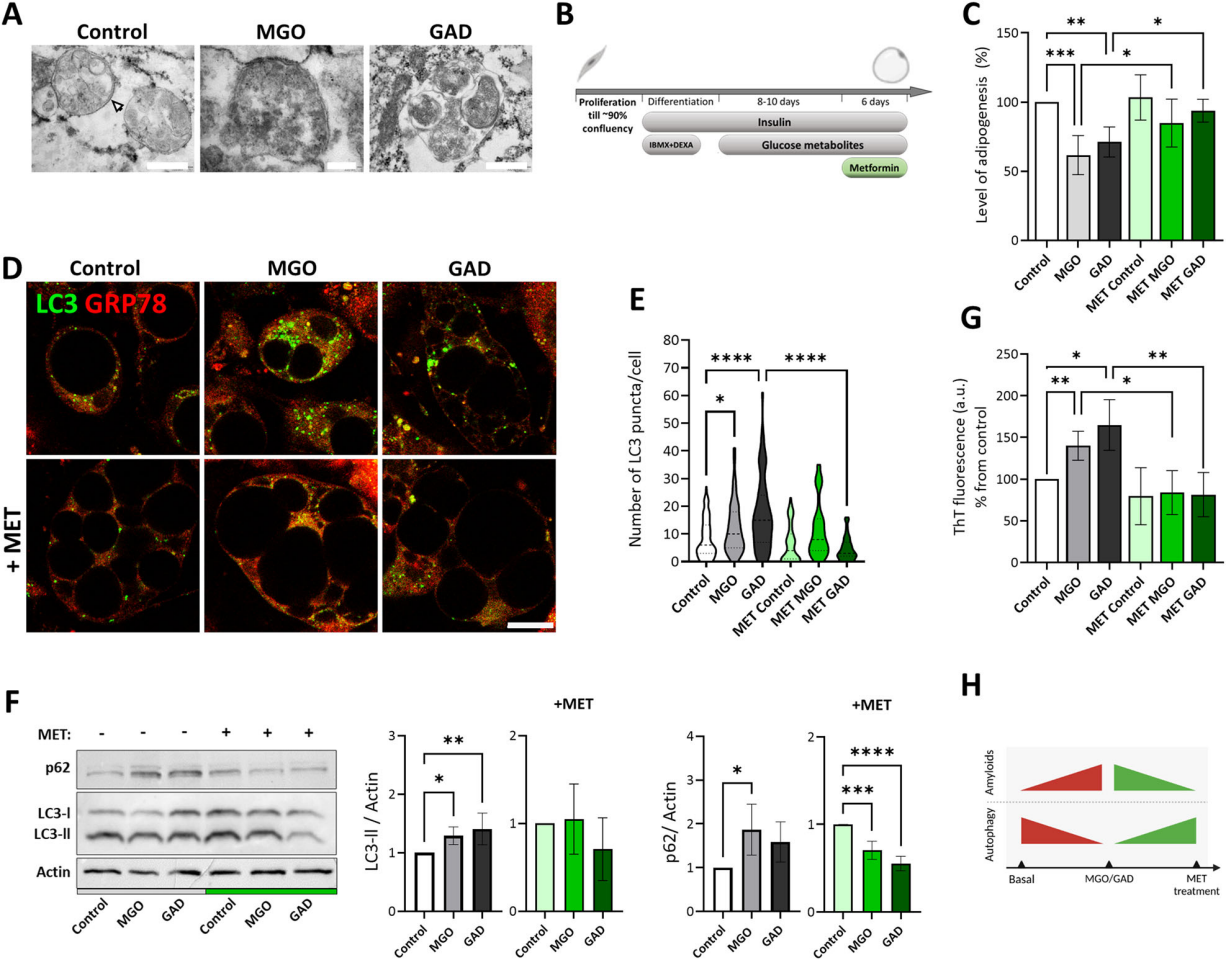
Metformin impact on autophagy in MGO and GAD treated adipocytes. **A** TEM images of autophagy vesicles formed upon treatment with MGO and GAD. White arrow points to the autophagosome double membrane (Magnification and scale bar: control- ×50k, 500 µm; MGO- ×100k, 200 µm; GAD- ×60k, 500 µm). **B** Schematic illustration of experimental timeline with metformin (MET) treatment. **C** LOA quantification of MGO and GAD treated adipocytes compared to controls (*N* = 6). Green coloring represents MET treated groups. **D** Confocal images of autophagy puncta stained with anti-LC3 in MGO/GAD treated adipocyte cells with MET treatment (Magnification of ×630, scale bar = 20 µm). **E** Quantification of LC3 puncta number per cell in treated adipocytes (control, *N* = 89; MGO, *N* = 57; GAD, *N* = 68; MET, *N* = 96; MET + MGO, *N* = 59; MET + GAD, *N* = 85 cells). **F** P62 and LC3 II analyzed protein expression levels in adipocytes treated with MGO, GAD, and with (green coloring) or without MET (LC3 II: *N* = 5; p62: *N* = 4). **G** ThT fluorescent spectroscopy of MGO/GAD and MET treated adipocytes cell lysates (*N* = 6). Data are presented as mean ± SD. One-way ANOVA with Tukey’s post hoc test, (ns) *p* > 0.05; *p < 0.05; **p < 0.01; ***p < 0.001; ****p < 0.0001. **H** Schematic summary of MET effect on autophagy and amyloid levels in MGO and GAD treated adipocytes.

Conformational changes, such as the formation of β-sheets, can cause proteins to become amyloidogenic, which is known to alter protein function. Such a protein becomes cytotoxic and must be eliminated/degraded to maintain proper cellular activity. The removal of misfolded proteins is accomplished by the autophagy pathway. TEM images of the MGO/GAD treated cells (Fig. 5A) reveal that the double membrane structure of the auto-phagosome (white arrow) that surrounds the cargo is dedicated to lysosomal degradation, while vesicles in cells grown with GAD appear larger. To better understand these observations, cultures were treated for 6 days with 5 μM MET, which is an inducer of autophagy (Fig. 5B).

We have previously reported an altered LOA in cells treated with MGO/GAD [26]. This was extended here to an examination of the effects of the carbonyl compound on lipolysis (Fig. 2), the formation of amyloids by imaging ThT^+^ LDs (Fig. 3), and by RAMAN spectroscopy (Fig. 4). The results indicated that treatment with MET could reverse the changes in adipogenesis seen in MGO/GAD treated cultures (Fig. 5C).

The autophagy process was monitored by staining for LC3 II expression, a marker protein for auto-phagosome vesicles and provides the outcome in a puncta assay. This study presents the first observation of autophagy in carbonyl compound treated adipocytes. We analyzed the increase in puncta (auto-phagosomes) at the single-cell level, which were co-stained with GRP78. The results revealed that the number of autophagy puncta is greater in cells treated with MGO/GAD than the values in control cells, with increases from 8.32 ± 6.99 LC3 puncta/cell (*N* = 89) in the control to 12.38 ± 9.3 (*N* = 57) and 17.28 ± 13.11 (*N* = 68) after treatment with MGO and GAD respectively (Fig. 5E). These results indicate that exposure to carbonyl compounds impairs the efficiency of the elimination pathways i.e., autophagy.

Therefore, it is interesting that the addition of MET significantly decreased the number of LC3 puncta that was quantified in the GAD treated cells by a factor of 3.7 and restored the values to control levels (MET + GAD: 4.69 ± 4.24, *N* = 85; GAD: 17.28 ± 13.11, *N* = 68; control 8.32 ± 6.99, *N* = 89). The results of cultures treated with only MET (6.45 ± 6.64 LC3 puncta/cell, *N* = 96) were like control group (Fig. 5D, E). The immune staining and punctate measurements indicate that MET improves the autophagy function under glycated conditions. These results were verified by western blot (WB) analysis of the autophagy process efficiency based on LC3 I/II and p62. Complementary to the autophagosomes quantification in Fig. 5D, E, there is an elevated level in LC3 expression following MGO/GAD addition while the addition of MET to cultures revers its levels (Fig. 5F). Additionally, p62 expression indicate an improvement in punctate clearance following the MET treatment (Fig. 5F). These findings confirm the puncta analysis results and support the hypothesis that AGEs formation affects autophagy and impairs the efficiency of the elimination pathways (Fig. 5E). Using spectroscopy, we were able to quantify the protein misfolding in cell lysates in the presence of ThT. Results indicate elevated levels of ThT fluorescence in the presence of MGO and GAD, which reflect increased amyloidogenic protein formation, correlated with impaired autophagy. The findings that in the presence of carbonyl metabolites we monitored an elevation of amyloid levels by approximately 50% compared to the control, while MET treatment restores control values (Fig. 5G–H), demonstrate that hyperglycemic conditions reduce the efficiency of autophagy and elimination of amyloid aggregates while MET restores this function.

### Amyloid formation in adipose tissue

**Fig. 6:**
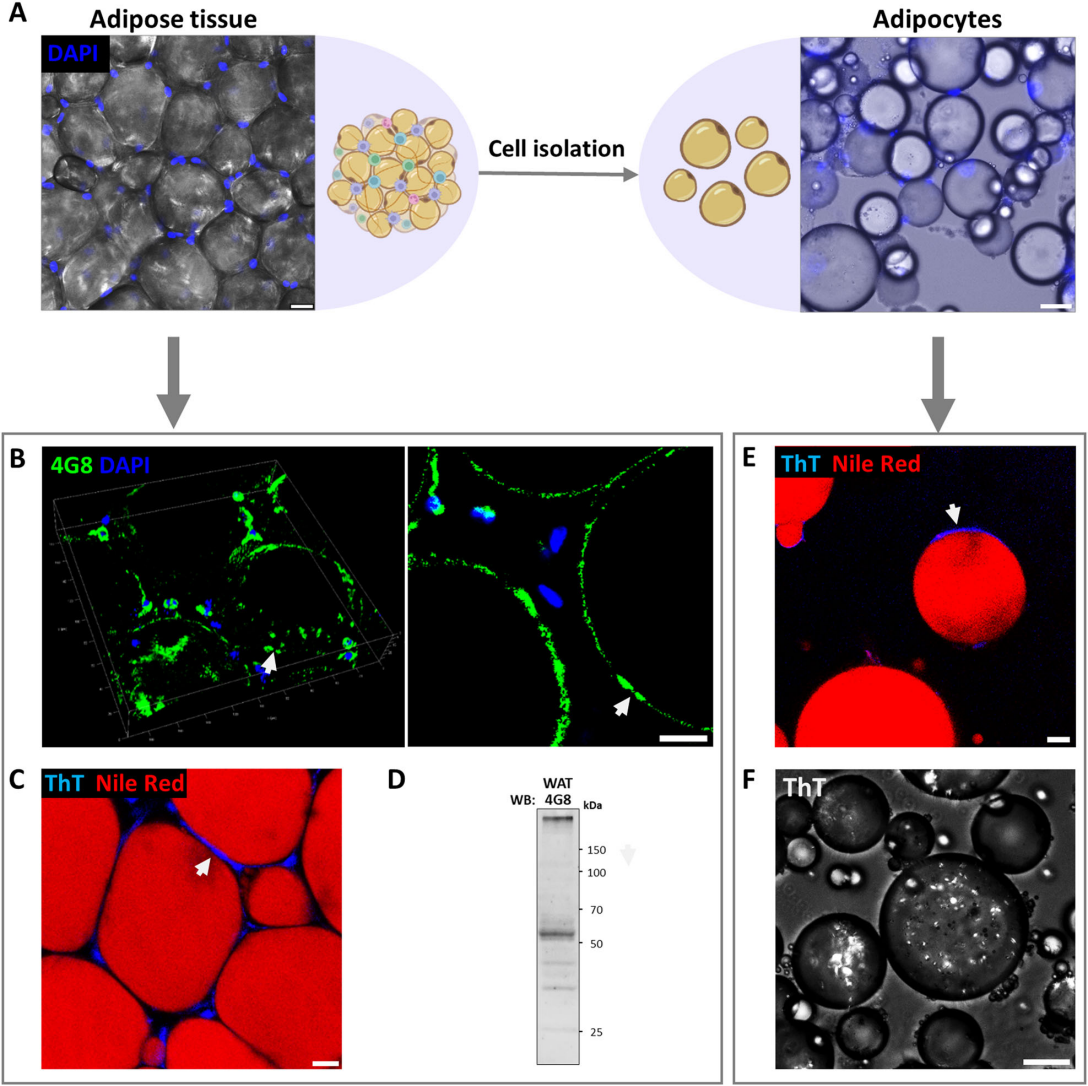
Lipid droplet associated protein aggregation in mouse adipose tissue and isolated adipocytes. **A** Adipose tissue (WAT) and primary adipocytes visualized before and after cells isolation (Scale bar: adipose tissue = 20 µm, adipocytes = 50 µm). **B** 3D confocal image (left image) of lipid droplet associated aggregates (white arrows), stained by 4G8 antibody. The arrows mark the same region on the LD surface in both images. (WAT; Magnification of ×200; Scale bar = 15 µm). **C** Lipid droplet associated aggregates (white arrow) visualized by ThT staining (blue) and lipid droplets stained by Nile-Red. (WAT; Magnification of ×200; Scale bar = 15 µm). **D** Immunoblotting of WAT lysate with 4G8 antibody. **E** Fluorescence microscopy of isolated primary adipocytes stained with ThT (amyloids, white arrow) and Nile-Red (lipid content) (Magnification x200; Scale bar = 15 µm). **F** Polarized light microscopy of ThT stained isolated adipocytes (Scale bar = 60 µm).

**Fig. 7:**
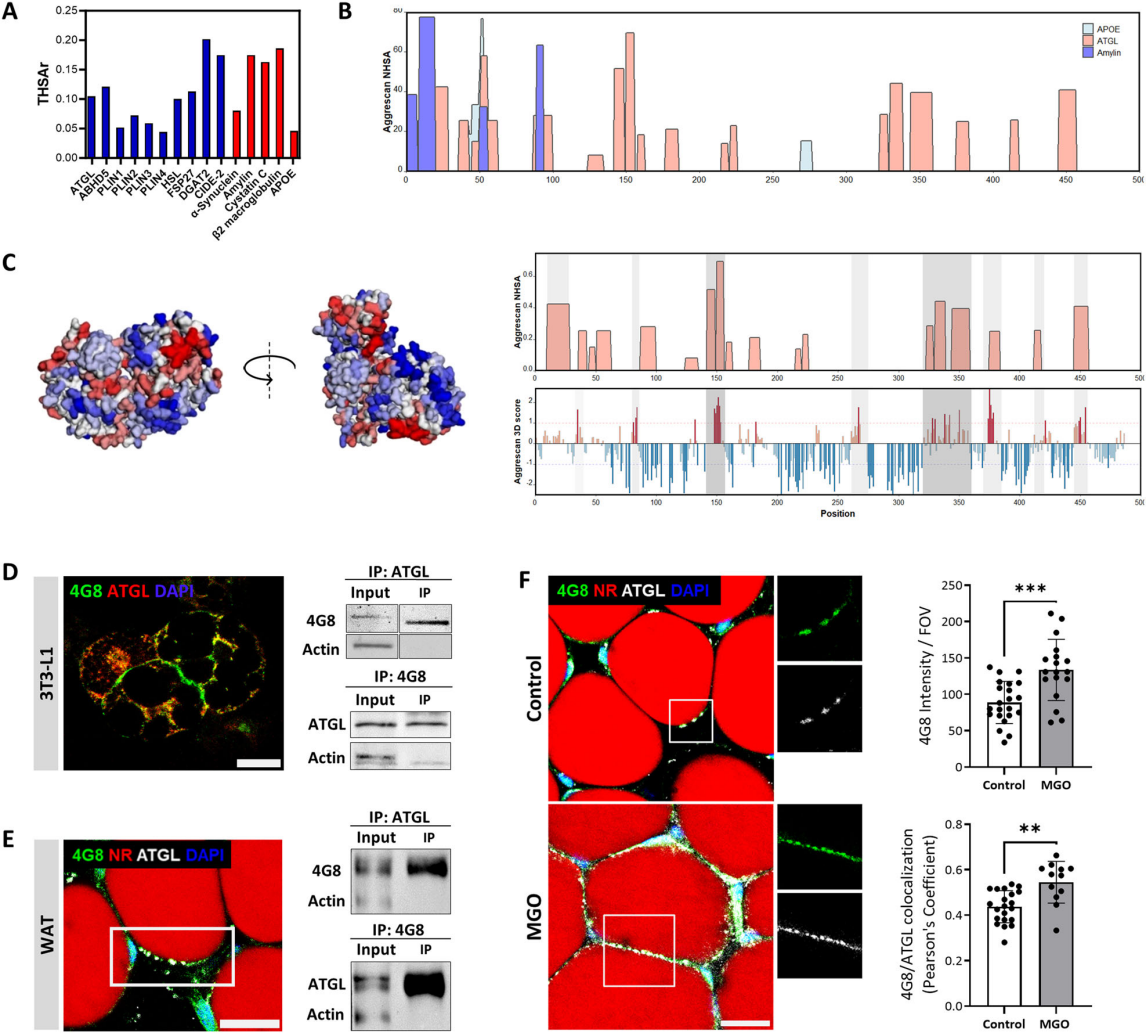
Prediction of lipid droplet associated protein that promotes amyloid secondary structure. **A** AGGRESCAN THSAr score for protein amyloid aggregation of lipid droplet-associated (blue) compared with known amyloid forming proteins (red). **B** The protein sequences of ATGL (red), APOE (light blue), and Amylin (blue) plotted by the AGGRESCAN aggregation “hot spots” tool. **C** 3D AGGRESCAN score, alignment correlation of susceptible regions for amyloid formation in 2D and 3D schemes of ATGL protein. **D** IF co-staining of ATGL and 4G8 antibodies in 3T3-L1 adipocytes (left; magnification of ×630; Scale bar = 20 µm). Co-immunoprecipitation of ATGL and 4G8 and WB with 4G8 and ATGL respectively (right). **E** IF staining in WAT for ATGL and 4G8 (left; magnification of ×200; Scale bar = 20 µm). Co-immunoprecipitation of ATGL and 4G8 and WB with 4G8 or ATGL in WAT protein extraction (right). **F** IF co-staining for ATGL, 4G8 and Nile-Red in control and MGO-treated WAT (left; magnification of X200; Scale bar = 20 µm). 4G8 intensity and 4G8/ATGL co-localization quantification in control and MGO-treated WAT adipocytes (N: control = 4, MGO = 3; right panel). Data are presented as mean ± SD, FOV- field of view. Student’s t-test; **p < 0.01; ***p < 0.001.

The protein misfolding associated with LDs was observed in cell cultures and was validated by multiple assays. We also demonstrated the appearance of amyloid proteins associated with LDs membrane in white adipose tissue (WAT; Fig. 6B, C) and freshly isolated mature adipocytes (Fig. 6E, F) using 4G8 antibody on IF staining, or ThT staining visualized by confocal and polarized microscopies. To co-localize the alteration to a specific protein, we performed a WB with the 4G8 antibody on protein extracted from the WAT (Fig. 6D) revealed two bands of 55 kDa and >150 kDa, the higher band suggested for protein aggregates that was further confirmed by immunoprecipitation (Fig. 7) and MS/MS results as ATGL. Complementary genomic results indicate that ATGL is differentially expressed in adipocytes in mouse and human adipose tissue and in differentiated 3T3L1 cells (Figure S1).Using bioinformatics tools were utilized to identify the LDs’ membrane proteins responsible for amyloidogenic alteration in proteins structure. Protein alterations rely on their amino acid sequences as not all proteins become amyloidogenic. The candidate proteins related to LDs that potentially undergo misfolding were screened for aggregation-prone regions. AGGRESCAN tool allows to predict “hot-spot” areas along the protein sequence with the potential for amyloid aggregation. Comparison of the THSAr scores of LDs related proteins to known amyloidogenic proteins (Fig. 7A), revealed that 6 out of the top 10 LDs proteins, namely ATGL, ABHD5, HSL, FSP27, DGAT2, and CIDE-A have a high amyloidogenic score ( > 0.10). Taking this result together with the molecular size of the protein detected by the western blot (Fig. 6D), it suggests that the observed protein is of a 55 kDa size, thus identifying the candidate protein as ATGL monomer (Fig. 7A). The hot spot profile of ATGL is presented alongside amylin and APOE, which are known to form amyloid structures, with high score for amyloid formation in specific regions (Fig. 7B). In relation to ATGL protein structure, comparison of 2D and 3D AGGRESCAN scores indicates a considerable overlap of the amyloidogenic regions predicted by these methods. 3D AGGRESCAN revealed several regions in the ATGL sequence that are prone to amyloid formation, namely located around 140–160 and 330–360 AA have a particularly high correlation with the 2D and 3D prediction and are thought to affect the protein 3D structure (Fig. 7C). Additionally, prediction tools PASTA-2, Fold Amyloid, MetAmyl, and WALTZ also confirm the identified sequence regions repeatedly emerge as hot-spots for amyloid formation (Fig S3B).

In Fig S3c, STRING network analysis allows to demonstrate the linkage between amyloids, autophagy, and LD-related proteins. The presented proteins interaction network reveals that the proteins are clustered into four groups with associations with: lipid droplets, adenosine monophosphate-activated protein kinase (AMPK), autophagy, or mTOR signaling and there are functional connections between these groups. For example, the insulin receptor (Insr) signaling pathway (Irs1/IRS1-insulin receptor substrate) is connected to the autophagy through p62 (SQSTM1) that is an adaptor protein for selective autophagy of ubiquitinated protein aggregates and is commonly used to assess the autophagy flux (Fig. 5). P62 is also correlated with the degradation of amyloid aggregates because of the formation of misfolded proteins that p62 can recognize. In these networks, ATGL (Pnpla2), is a prominent protein of the lipid droplet cluster, and STRING analysis connects this protein to diabetes related pathways including mTOR signaling and amyloid formation.

Expanding on these findings, we further explored the role of autophagy in ATGL elimination. We applied immunoprecipitation for LC3 followed by a western blot analysis for ATGL. Our findings confirmed the interaction between ATGL and LC3, which was enhanced in the presence of MGO/GAD, providing evidence that glycation hampers autophagy (Fig. S2). This suggests that the glycation impairs the autophagy process and leads to the accumulation of ATGL within autophagosomes. These results complement the immunofluorescence data showing punctate accumulation (Fig. 5D, E).

The results of the bioinformatics (Fig. 7, S3) and western-blot analyses (Fig. 6D) led us to focus on ATGL as the candidate protein for the amyloidogenic alterations observed on the LDs. Therefore, we used the 4G8 antibody, recognizes amyloid structures in other proteins, to confirm the amyloid nature of ATGL, as demonstrated by IF and CO-IP of ATGL and 4G8 (Fig. 7D, E). The co-staining of ATGL and 4G8 in cultured adipocytes (Fig. 7D) and WAT (Fig. 7E) result with co-localization, overlap regions suggest the ATGL conformational changes. The result was strengthened by CO-IP and WB assays with 4G8 antibody that was positive to ATGL and inversely, in both cell culture and WAT (Fig. 7D, E). In this regard we employed the IP of proteins from adipose tissue using the 4G8 antibody that were separated on SDS-gel followed by in-gel protein digestion (based on protein size) to further employed the MS/MS analysis. Combining the IP and MS/MS allows for the evaluation of the protein glycation and amyloidosis from numerous recovered proteolytic cleavage peptides and the MS/MS identification of ATGL sequence. The ATGL sequences possess numerous Lysin (K, 15 sites) and Arginine (R, 30 sites) sites that are candidates for glycation (Fig S3a). Several peptides, isolated by MS/MS, identified the ATGL including hot spots for glycation sites following the IP. The peptides localized to amino acids 164–179 and 289–294 were un-glycated, while other peptides were glycated and corresponded to 385–394 (R385; K386) and 447–464 (R465), retrieved from the 55 kDa isolated protein (monomer). Peptides recovered from the 150 kDa band are corresponding to the ATGL sequence, indicating that this protein forms aggregates and AGEs, with glycated spots corresponding to 110–122 (R114, R121), 208–224 (R220), and 283–265 (R293, R297, K300) amino acids.

We also have shown the MGO enhanced AGEs formation in adipose tissue in vivo, where mice were treated with 0.5% MGO in drinking water for 15 weeks. Following the treatment, immunostaining for co-expression of ATGL and β-amyloid structures (using the 4G8 antibody) was visualized on the lipid droplets of WAT and quantified (Fig. 7F). The results showed that MGO treatment significantly increased the formation of amyloids and confirmed the amyloidogenic appearance of ATGL on the lipid droplets of adipocytes in vivo.

In this study, we present novel findings that shed light on ATGL, a key protein in the structure and function of the lipid droplets, which becomes associated with the formation of β-amyloid secondary structure that is enhance under glycation. This discovery unveils a previously unknown link between ATGL and amyloidogenic protein changes in adipose tissue function, offering valuable insight into potential mechanisms underlying diabetes-related complications and serve as a new platform that confirms the hypothesis of protein glycation in T2D.

## Discussion

Exposure to hyperglycemia leads to insulin resistance and metabolic impairment. Prolonged hyperglycemia results in the formation of glucose carbonyl metabolites and subsequently to the development of AGEs through protein glycation. Carbonyl metabolites, such as MGO and GAD, cause protein misfolding and structural changes that can affect protein functionality [15, 33, 34]. Since adipocytes are central players in glucose metabolism, they were selected as a model system to study the effect of carbonyl compounds on protein alterations, and the deposition of β-amyloid structures formation. To this end, we examined the formation of amyloid formed proteins on LDs in adipocytes cells in the presence of MGO and GAD and in WAT. We also examined the effect on autophagy to understand whether the observed accumulation of misfolded glycated proteins can be attributed to a failure of the physiological elimination mechanisms.

Metformin (MET), prescribed as a first-line treatment for T2D, has also been shown to decrease serum levels of MGO by about 30% in humans [35]. MET is thought to influence the MGO level through a direct chemical interaction between the two molecules (MGO-MET), or MET effect on glycemic control that inhibit MGO production [36, 37]. The effect of MET on GAD treated cells has so far been reported only in-vitro study on macrophage cell line. In other aspect, the effect of GAD on LDL (low-density lipoprotein) glycation leads to cholesterol accumulation in macrophages and this process was inhibited by MET [38]. Previous studies describe the effect of MET on adipocyte differentiation, and regulation of ER-stress and AMPK signaling [39–41]. The results present the beneficial impact of MET on autophagy in MGO and GAD treated adipocytes, autophagy affected by AMPK signaling (Fig. 5). In addition, MET restores the level of adipogenesis after this has been reduced when exposed to MGO and GAD and also lowering cellular amyloid level that were elevated by the carbonyl compounds (Fig. 5G). In this study, profound effects were observed using low concentrations of MET (5 µM), whereas other research involving 3T3-L1 cells required higher doses (1.25–2.5 mM) to impact adipogenesis and AMPK signaling pathways [39, 40]. As suggested in Fig S3c, the STRING network connected the AMPK role on diverse cellular processes including autophagy, mTOR signaling, as well as a correlation with autophagy and lipolysis. mTOR is connected to the hexokinase 2 enzyme (Hk2), which phosphorylates glucose to produce glucose-6-phosphate for glycolysis and is upregulated in T2D.

Our data uncover the effect of AGEs formed in 3T3-L1 cells on molecular vibrations, as observed in the RAMAN spectra peaks that reflect secondary structures. The RAMAN spectroscopy is a sensitive technique that provides a biochemical “fingerprint” to resolve the structural molecular changes in the intracellular compartment [42, 43]. We identify the changes in the intensity of the amide I and III peaks as a response to hyperglycemia in adipocytes. These changes reflect alterations in protein conformation and an increase in β-sheet structures (Fig. 4). Such changes in the 1655 and 1260 cm-1 peaks were previously described in primary mice neurons after exposure to amyloid- β protein oligomers [44], and β-sheet formed protein accumulation in pancreatic cancer cells [45]. The RAMAN results are well correlated with the ThT spectroscopy (Fig. 5G), staining and polarized microscopy (Fig. 3) that emphasize the LD-associated amyloid aggregations.

Lipid droplet-associated proteins are a group of proteins that have been shown to be critical in regulating lipid metabolism. Among this group, ATGL has emerged as a central player in the regulation of lipolysis and triglyceride (TG) metabolism. ATGL has been shown to be essential for efficient lipid mobilization from lipid droplets, particularly in response to energy demands [46, 47]. In addition, recent studies have demonstrated that ATGL may also be involved in the regulation of autophagy-mediated lipid degradation by the LC3-interacting region (LIR) motif [48]. Our results demonstrate the negative effect of AGEs on both lipolysis and autophagy (Figs. 2, 5 and S2). Interactions between LDs associated proteins and autophagy-related proteins suggest these proteins play a critical role in maintaining cellular lipid homeostasis. ATGL knockout mice, which lack this key player in the lipolysis process, tend to exhibit moderate obesity [49, 50]. This suggested to be correlated with the impaired lipolysis observed in adipocytes following treatment with MGO and GAD, accompanied by an elevation in the formation of amyloid structures associated with lipid droplets (Figs. 2, 3). Our findings underline the significant role of ATGL and suggest that the disruption of its function could be intensified by the formation of β-amyloid structures on proteins associated with lipid droplets (Figs. 6, 7) as shown by immunostaining, western blot and MS/MS.

Amyloid-forming proteins, like amylin in the pancreas, play a role in the development of type 2 diabetes (T2D) when they form amyloid fibrils [51–53]. These amyloid deposits accumulate in the pancreas, thereby impairing β cell function and disrupting glucose homeostasis. Interestingly, there is a high comorbidity between diabetes and other amyloid-related diseases, such as Alzheimer’s disease and systemic amyloidosis [54–56]. This suggests the presence of common mechanisms and processes involving amyloid formation. Understanding these mechanisms is crucial for developing treatments for amyloid-related diseases. In the context of T2D and amyloidosis, we propose to explore adipocytes as a novel focal point for investigation.

In summary, the results of the current study highlight the detrimental effects of hyperglycemia-induced glucose carbonyl metabolites and AGEs formation on adipocyte differentiation and metabolism. Such condition promotes protein misfolding, leading to the formation of β-amyloid deposits and protein aggregation, making them more difficult to remove due to autophagy dysfunction. Furthermore, our results suggest that targeting lipid droplet-associated proteins, such as ATGL, may hold promise for developing therapeutic interventions for metabolic disorders. The presented findings could potentially serve as valuable markers for gaining deeper insights into metabolic changes occurring in adipose tissue and is a significant step forward in understanding the role of ATGL in cellular dysfunction, investigating the interplay between AGEs formation, amyloidosis and T2D. Further research in this area is necessary to unravel the underlying mechanisms and explore potential treatment strategies for amyloid-related diseases.

## Materials and Methods

### Visceral adipose tissues (VAT)

Epididymal white visceral adipose tissue (WAT) were collected from C57BL/6 J mice (Envigo RMS Limited, Jerusalem, Israel) and immediately used as fresh, frozen (by liquid nitrogen) or for mature adipocyte isolation following collagenase digestion as previously described [57, 58]. The mice were kept in a conventional facility of Tel Aviv University (TAU) with 12 h light/dark cycles and were fed with standard chow diet and water were provided ad libitum. Methylglyoxal (MGO) treated mice: Male 6-wk-old C57BL/6 J mice were given regular drinking water or, in MGO group, water supplemented with MGO (M0252, Sigma-Aldrich) up to a final concentration of 0.5% for 15 weeks. Animal care and experiments were in accordance with the guidelines of the TAU- IACUC Approval (01-21-044). **Mature adipocytes:** Were isolated from WAT as described [57]. Briefly, the tissue was ground to a fine consistency in HBSS solution (Biological Industries, Israel), then incubated with collagenase solution (Sigma-Aldrich, c-5138) for one hour at 37^◦^C with shaking. The digested tissue was filtered through a 100 µm cell strainer (SPL life science), centrifuged at 1800 rpm for 5 min and mature adipocytes (floating fraction in the isolation medium) were collected.

### Cell culture, adipogenic differentiation and hyperglycemia cell growth

3T3L1 cells (ATCC, USA) were seeded at 1 × 10^4^ cells/cm^2^ in a growth medium (GM, Dulbecco’s modified Eagle’s medium DMEM, 4.5 mg/ml glucose; Gibco™) with 10% fetal bovine serum and 1% L‐glutamine (Biological Industries), 0.1% penicillin–streptomycin (P3032, 85555; Sigma‐Aldrich), 0.5% 4‐(2‐hydroxyethyl)‐1‐piperazinee thane-sulfonic acid (HEPES; Biological Industries). Differentiation medium (DM) supplemented with 100 IU/mL insulin (41-975-100; Biological Industries, Israel), 1 μM dexamethasone (Sigma‐Aldrich), and 400 μM 3-isobutyl-1-methylxanthine (IBMX; 2885842; BioGems) for two days then replaced by a supporting medium (SM); consisting of the GM supplemented with 100 IU/mL insulin [59, 60]. Once adipogenesis was visualized in the cells, carbonyl compounds - methylglyoxal (MGO, M0252) or glycolaldehyde (GAD, G6805) both from Sigma-Aldrich were added to the medium in a final concentration of 0.001 mg/ml or 0.01 mg/ml, respectively for 14 days and in some cultures 5 µM metformin (MET) (BioVision, 1691) added for a period of 6 days. The cultures incubated at 37 °C in humidified atmosphere containing 5% CO_2_.

### Morphological features to follow differentiation

Cells growth and differentiation process was followed by morphology changes, LDs accumulation monitored on live cultures. The adipogenesis quantification at the macro-scale ×40 for mapping the level of adipogenesis (LOA) follow up using phase-contrast EVOS FL Auto-2 Microscope (Invitrogen) [61]. Images at higher magnification x200 allow to analyze the cell and LDs size [60].

### Bioinformatics

The functional connection between proteins analyzed by STRING software https://string-db.org/ site [62]. Cytoscape software platform used for visualizing the STRING networks [63]. Relative expression of the ATGL gene was analyzed in different WAT cell populations using scRNA-seq datasets for both mouse and human from the Single Cell Portal (SCP1376) [64]. Mass spectrometry data of cultured adipocytes were analyzed based on previously described data [60].

### In silico aggregation analysis

AGGRESCAN server (http://bioinf.uab.es/aggrescan/) [65] was employed to predict hot-spot aggregation regions in various LD-bound proteins. The server utilizes an algorithm that assesses the potential for amyloid aggregation based on the input protein sequence. Their THSAr (Total hot spot area per residue) score was calculated to quantitatively evaluate the amyloidogenic potential of the target proteins. This score provides an indication of the protein’s propensity for amyloid aggregation. AGGRESCAN 3D server was used to predict potential aggregation regions of ATGL (http://biocomp.chem.uw.edu.pl/A3D2/) [66]. This tool allows the visualization of predicted hot-spot aggregation regions in three dimensions. Complementary aggregation property predictions were also performed using the WALTZ (https://waltz.switchlab.org/index.cgi), PASTA2 [67], MetAmyl [68], and FoldAmyloid [69] algorithms that focus on amyloidogenic regions predictions of ATGL sequence (Pnpla2 protein [Mus musculus]; Accession: UniProt Q8BJ56-1).

### Cell morphology, staining and imaging

#### Whole-Mount immunofluorescence staining

VAT whole-mount staining was performed as previously described [59]. Primary antibodies: ATGL (mouse, Santa Cruz sc-365278), 4G8 (Beta Amyloid 17-24; mouse, Bio Legend, SIG-39240). Secondary antibodies: Alexa 488 (A 21141), Alexa 555 (A-21127) from Invitrogen, mounting medium Fluoroshield™ containing 4′, 6-diamidino-2-phenylindole (DAPI) (Electron Microscopy Sciences, #17985-10) and viewed by confocal microscope (Leica SP8; Leica, Wetzlar, Germany).

#### Immuno-fluorescence (IF) cell staining

Cells were fixed using 4% paraformaldehyde containing 0.03 M sucrose in phosphate-buffered saline (PBS) for 10 min at RT, then washed in 1% PBS with 0.5% triton. Blocking solution, Tris buffer saline (TBS) 1% containing 1% normal goat serum and 1% bovine serum albumin (BSA). The cells incubated with primary antibodies: LC3b (rabbit, Abcam ab192890; mouse, Sc-271625), GRP78 (rat; SC-13539), perilipin 1 (mouse; SC-390169), GLUT4 (mouse; SC-53566), ATGL (mouse; Santa Cruz SC-365278), 4G8 (Beta Amyloid 17–24; mouse, Bio Legend, SIG-39240). Secondary antibodies; Anti-mouse: Alexa 488 (A21121 and A 21141), Alexa 555 (A-21127) from Invitrogen, Anti-rabbit: Cy3 (Jackson Immuno Research Laboratories, Inc.), Anti-rat: donkey anti-rat Alexa 647 (Invitrogen). The stained cells were mounted with fluoro-Gel mounting medium containing 4‘, 6‐diamidino‐2‐phenylindole (DAPI) (17985-50, Electron Microscopy Sciences). Cells visualized and photographed by fluorescence microscopy (Nikon, Eclipse Ci) or by Zeiss LSM-710 confocal microscope and analyzed using ImageJ software (NIH, Bethesda, MD, USA).

#### Glucose uptake assay

using a fluorescent glucose analog, 2-NBDG (2-Deoxy-2-[(7-nitro-2,1,3-benzoxadiazol-4-yl) amino]-D-glucose) (11046, Cayman chemical). Cultured 3T3-L1 cells “starved” in a glucose-free medium at 37 °C for 1 h, then added 100 µM 2-NBDG and insulin for 1 h, washed with PBS and fresh media was added, cultures observed by live imaging (Incucyte® SX5, Sartorius).

#### Fluorescent staining for amyloids and lipid droplets

Fixed adipose tissue, isolated adipocytes, or cultured adipocytes were incubated with 5 mM Thioflavin T (ThT; Sigma T3516), 7 mM Congo red (CR; T6277, Sigma) and 10 µg/ml Nile Red (Sigma N-3013) for 30 min in room temperature, then were mounted with fluoroshield mounting Fluoroshield™ (Electron Microscopy Sciences, #17985-10, without DAPI), images were acquired by confocal microscope (Leica SP8; Leica, Wetzlar, Germany). ThT and CR-stained cells observed, and images acquired using polarized light microscope (Nikon, Japan), fluorescence microscope (Eclipse Ci; Nikon), EVOS FL Auto-2 Microscope (Invitrogen), confocal microscope- Zeiss LSM-710, Chameleon 720 (690-1064) laser and images were analyzed by ImageJ.

#### Transmission Electron Microscopy (TEM)

Cells were fixed overnight in 2.5% Glutaraldehyde in phosphate-buffered (PBS) at 4 °C was then washed several times with PBS and post-fixed in 1% OsO4 in PBS for 2 h at 4 °C. Dehydration in graded ethanol and embedded in Glycid ether for preparation of thin sections mounted on Formvar/Carbon coated grids. Sections were stained with uranyl acetate and lead citrate and examined using a JEM 1400 Plus transmission electron microscope (Joel, Japan). Images were captured using SIS Mega view III and the TEM imaging platform (Olympus).

#### RAMAN microscopy and spectroscopy

Adipocytes were treated with MGO and GAD for 11 days and then cells were re-plated on 60 nm gold-coated glass coverslips for two days. For the RAMAN spectroscopy, cultures were washed with PBS and air-dried. Cells were excited with a 532 nm laser and the spectral range was 1000-1800 cm-1, with maximal laser power of 10 mW and exposure time of 60 s collected the RAMAN spectral recorded by Lab Ram HR of Horiba Jobin Yvon (NewRoad, Olympus, Japan). Images were acquired by objective ×50 (N.A = 0.55), and the spectra were collected by objective ×100 (N.A = 0.9) (Olympus, Japan). The RAMAN signal collected by the CCD detector cooled down to −70° and RAMAN spectra processing was obtained using SpyctraGryph software.

#### ThT spectroscopy

Cells harvested from cultures were lysate in 25 mM Tris pH 7.4, 150 mM NaCl buffer containing 0.5% sodium deoxycholate, 0.1% SDS, and protease inhibitors: (phenylmethylsulfonyl fluoride, PMSF 1 mM; 1‐chloro‐3‐tosylamido‐4‐phenyl‐2‐butanone, TPCK, 10 μg/ml; aprotinin, 10 μg/ml Sigma) incubated for 30 min on ice. Thioflavin T (ThT; T3516, Sigma-Aldrich) 30 µM was added to samples in 96-well black microplate measured at excitation 420 nm/emission 485 nm using a Synergy™ H1 multi-mode microplate reader (BioTek Instruments, USA) [15].

#### Lipolysis

3T3‐L1 adipocytes cultured and treated with MGO and GAD as described. For the lipolysis induction, 10 μM of Forskolin (PeproTech) was added to the supporting medium; the forskolin was kept for 3 h. as described by us [31]. Live imaging and visualization were performed using EVOS FL Auto-2 Microscope (Invitrogen).

### Biochemistry and protein identification

#### Immunoprecipitation and western blot (WB)

Protein extraction or immunoprecipitation (IP) using Protein A/G PLUS- Agarose (Santa Cruz biotechnology; sc-2003) [59]. The protein samples were re-suspended in Lamelli buffer and separated on SDS polyacrylamide gel electrophoresis gel, then transferred to nitrocellulose. Nitrocellulose membranes incubated with primary antibodies against LC3b (rabbit, Abcam ab-192890), p62 (rabbit, Abcam ab-109012), ATGL (mouse, Santa Cruz sc-365278), 4G8 (mouse, Bio Legend, SIG-39240; 800720), HSP70 (mouse, Santa Cruz sc-7298), and actin (mouse, MP- 691001/2). For immunoprecipitation: LC3B (mouse, Cell Signaling #83506), ATGL (mouse, Santa Cruz sc-365278) or 4G8 (mouse, Bio Legend, SIG-39240; 800720), Secondary antibodies horseradish peroxidase (HRP) conjugated used; bovine anti-rabbit (Santa Cruz; sc-2370), goat anti-mouse (Jackson; 115035). Western blot chemiluminescent substrate (Super Signal^TM^ West Pico PLUS, 34578, Thermo Fisher) exposed and quantified digitally on Fusion FX7 (Vilber Lourmat).

#### Mass-Spectrometry analysis (MS/MS)

In-gel proteolysis and Mass-Spectrometry analysis- of cell lysate that was precipitated with anti 4G8 antibody (mouse, Bio Legend, SIG-800720), proteins were separated on SDS – gel and stained by Coomassie blue to retrieve the bands at the appropriate MW corresponding with the western blot analysis. The isolated gel lanes were reduced with 3 mM DTT (60 °C for 30 min), modified with 10 mM iodoacetamide in 100 mM ammonium bicarbonate (in the dark, room temperature for 30 min) and digested in 10% acetonitrile and 10 mM ammonium bicarbonate with modified trypsin (Promega) at a 1:10 enzyme-to-substrate ratio, overnight at 37 °C. The tryptic peptides were desalted using C18 tips (Homemade stage tips) dried and re-suspended in 0.1% Formic acid, 2% acetonitrile. The peptides were analyzed by LC-MS/MS using a Q Exactive HFX mass spectrometer (Thermo) fitted with a capillary HPLC. The peptides were loaded in solvent A (0.1% formic acid in water) on a homemade capillary column (30 cm, 75-micron ID) packed with Reprosil C18-Aqua (Dr. Maisch GmbH, Germany). The peptides mixture was resolved with a (5 to 28%) linear gradient of solvent B (100% acetonitrile with 0.1% formic acid) for 30 min followed by gradient of 15 min of 28 to 95% and 5 min at 95% acetonitrile with 0.1% formic acid in water at flow rates of 0.15 μl/min. Mass spectrometry was performed by in a positive mode (m/z 300–1500, resolution 60,000 for MS1 and 15,000 for MS2) using repetitively full MS scan followed by high collision dissociation (HCD, at 27 normalized collision energy) of the 18 most dominant ions ( > 1 charges) selected from the first MS scan. A dynamic exclusion list was enabled with exclusion duration of 20 s. The mass spectrometry data was analyzed using Protein Discoverer 2.4 (Thermo) using Sequest search engine, searching against the proteome from the mouse Uniprot database with mass tolerance of 8 ppm for the precursor masses and 0.02 amu for the fragment ions. Oxidation on methionine and protein N-terminus acetylation were accepted as variable modifications and carbamidomethyl on cysteine was accepted as static modifications. Analysis was added for semi-tryptic peptides with minimal peptide length was set to six amino acids and a maximum of two mis-cleavages was allowed for analysis of glycation options modifications on the ATGL (Pnpla2 protein; Accession: UniProt Q8BJ56). Minimal peptide length was set to six amino acids and a maximum of two mis-cleavages was allowed. The data was quantified by label free analysis using the same software. Protein analysis was performed at the Smoler Proteomics Center, Technion, Haifa, Israel.

#### Gene expression (qPCR)

As previously described [26], total RNA was extracted from 3T3-L1 cells using Trizol reagent (Bio Tri RNA; Bio-Lab Ltd., Jerusalem, Israel) and reverse transcribed to cDNA using a high-capacity cDNA reverse transcription kit (Applied Biosystem, Waltham, MA, USA). Transcript levels were measured with SYBR green (Applied Biosystem, Waltham, MA, USA) using STEPONE plus system (Thermo Fisher Scientific, Waltham, MA, USA). All data were normalized to actin by the delta-delta Ct method [70]. For qPCR amplification, we used the primers for the following genes:

Actin F-CATCGTGGGCCGCCCTAGGCACCA; R-CGGTTGGCCTTAGGGTTCAGGGGG

Glut4 F- TTCACGTTGGTCTCGGTGCT; R-TAGCTCATGGCTGGAACCCG.

ATGL (Pnpla2) F- GAGACCAAGTGGAACATC; R- GTAGATGTGAGTGGCGTT.

RPLP0 F-AGATTCGGGATATGCTGTTGGC; R- TCGGGTCCTAGACCAGTGTTC.

#### Statistical analysis

Statistical analysis was performed using GraphPad Prism 9 software (La Jolla, CA). One-way ANOVA or two-way ANOVA tests with Turkey post hoc test were performed in accordance with variable number, *p*-value < 0.05 was considered as statistically significant. All error bars shown in the figures are standard deviation (SD). Data plotted on GraphPad Prism 9 software and Microsoft Office Excel-2013.

#### Schematic illustrations

were created by the Bio Render software https://biorender.com.

## Supplementary information

Supplementary information

Supplementary information- WB full blots

Supplementary information- MS/MS

## Data Availability

All data generated or analyzed during this study are included in this published article.
