# Supplementary material for: Advanced glycation end-products accelerate amyloid deposits in adipocyte’s lipid droplets
Source: Cell Death Dis. 2024 Nov 19;15(11):846. doi: 10.1038/s41419-024-07211-6 (PMC11577098; doi:10.1038/s41419-024-07211-6)
Supplement: Supplementary file 1 — Supplementary information [file 41419_2024_7211_MOESM1_ESM.docx]

**Supplementary information**

**Advanced Glycation End-products accelerate amyloid deposits in adipocyte`s lipid droplets**

Roza Izgilov^1^, Nadav Kislev^1^, Eman Omari^1^, Dafna Benayahu *^1^

^1^Department of Cell and Developmental Biology, Faculty of Medical and Health Sciences,

Tel Aviv University, Tel Aviv 6997801, Israel


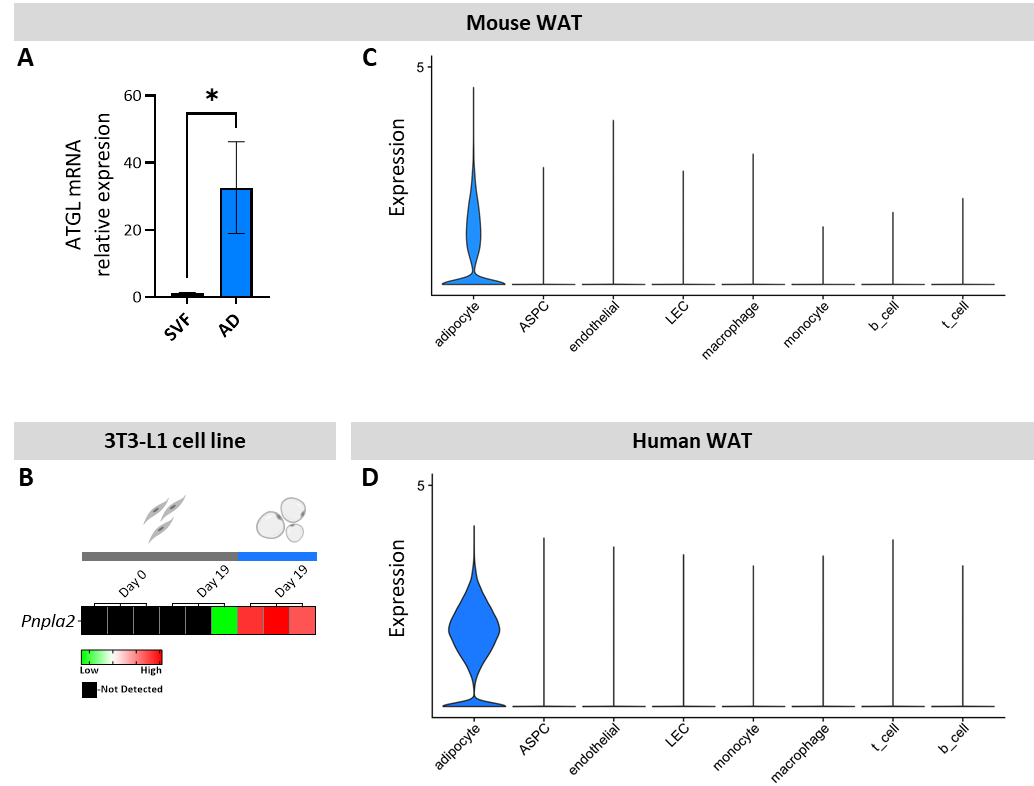


**Figure S1: ATGL expression in various adipose tissue cell populations.**

**A.** mRNA levels of ATGL in SVF (stromal vascular fraction) and AD (mature adipocytes) isolated from mouse WAT (Normalized to RPLP0, N = 3). Unpaired two- tailed t test, *p < 0.05. Data are presented as mean ± SD.

**B.** ATGL (PNPLA2) protein expression in 3T3-L1 pre-adipocytes and adipocytes at day 0 and day 19 of culturing from previous MS/MS analysis.

**C-D.** Relative expression of ATGL gene in different cell populations employing published WAT scRNA-seq analysis of mouse (**C**) and human (**D**) (Single Cell Portal - no. SCP1376).


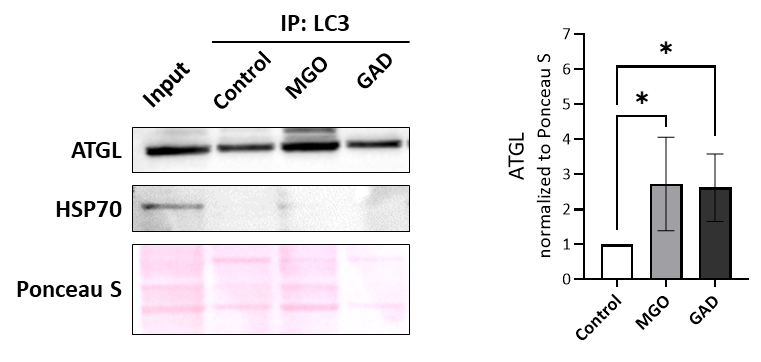


**Figure S2: ATGL interacts with LC3 as part of autophagy process.**

A representative co-expression of immunoprecipitation for LC3 and western blot of ATGL is enhanced by MGO/GAD in treated 3T3-L1 adipocytes. Quantification is normalized to Ponceau-S stained blot (N=4). Data are presented as mean ± SD. One-way ANOVA ; * p < 0.05.


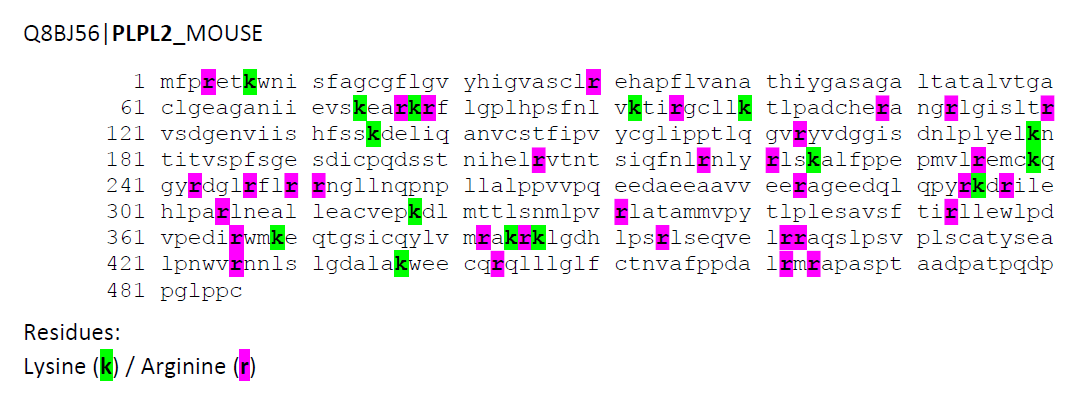

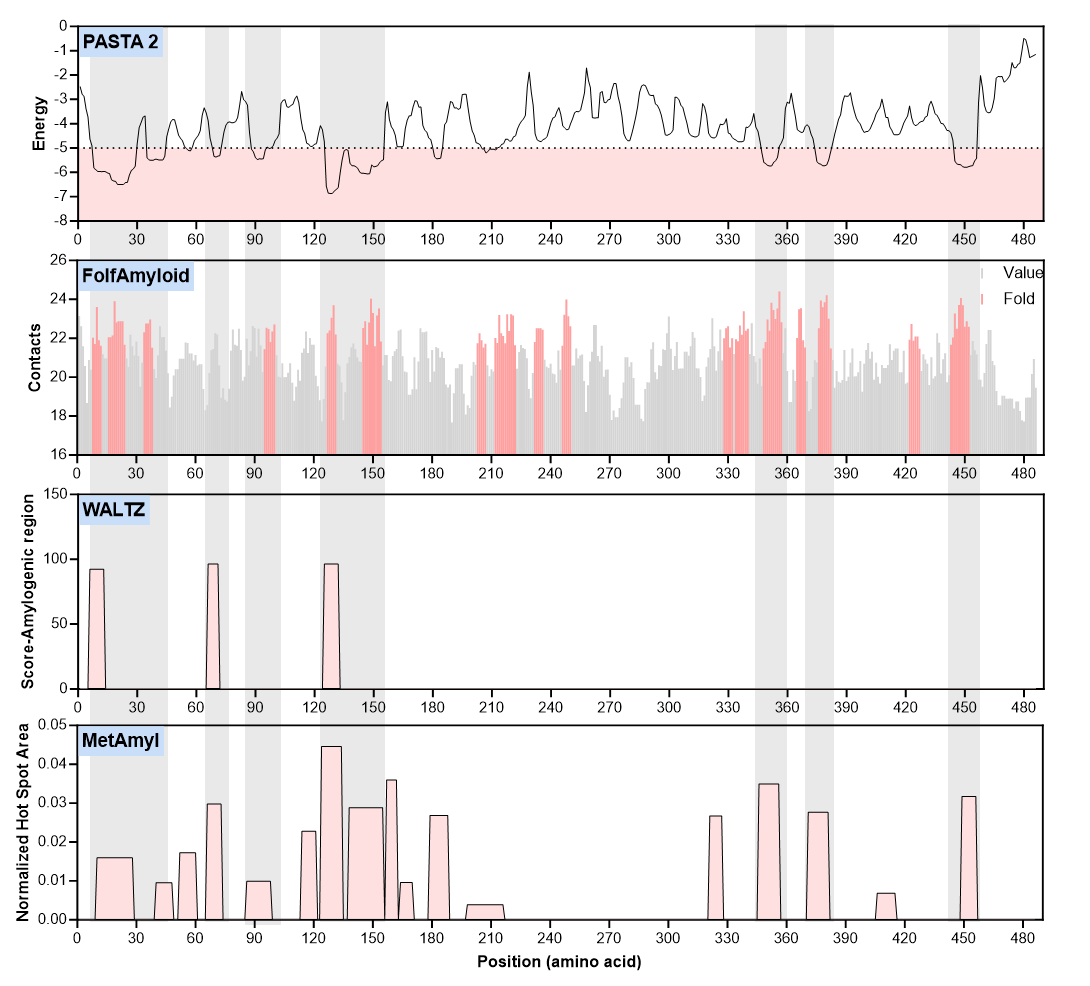

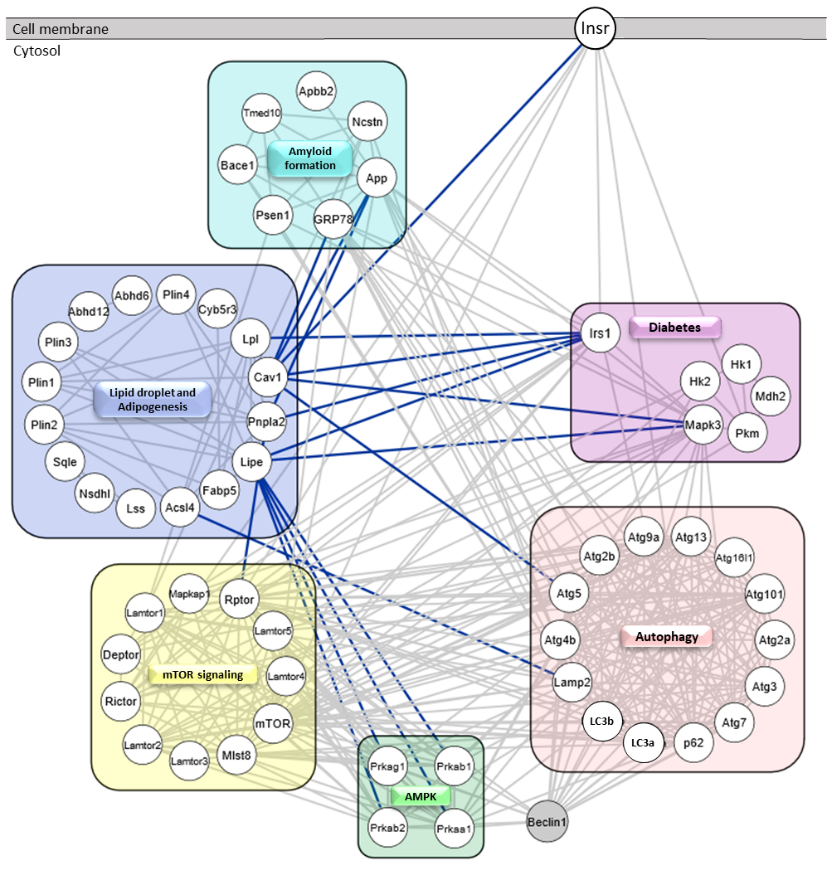


**B**

**C**

**A**

**Figure S3: ATGL amyloid propensity and protein interaction network.**

**A.** Sequence of the ATGL protein (Accession: Q8BJ56, mouse), with lysine (k, green) and arginine (r, purple) residues highlighted.

**B.** Predicted amyloid formation prone regions (red) of ATGL using PASTA 2 (energy<-5), FoldAmyloid, WALTZ and MetAmyl. Regions of the sequence that show correlation across three different algorithms are marked.

**C.** STRING proteins association network categorized by cellular function and location. The connection between “lipid droplet and adipogenesis” to other categories are marked in blue (Pnpla2 = ATGL).
