# Supplementary figures and images for: Advanced glycation end-products accelerate amyloid deposits in adipocyte’s lipid droplets
Source: Cell Death Dis. 2024 Nov 19;15(11):846. doi: 10.1038/s41419-024-07211-6 (PMC11577098; doi:10.1038/s41419-024-07211-6)

Figure 5

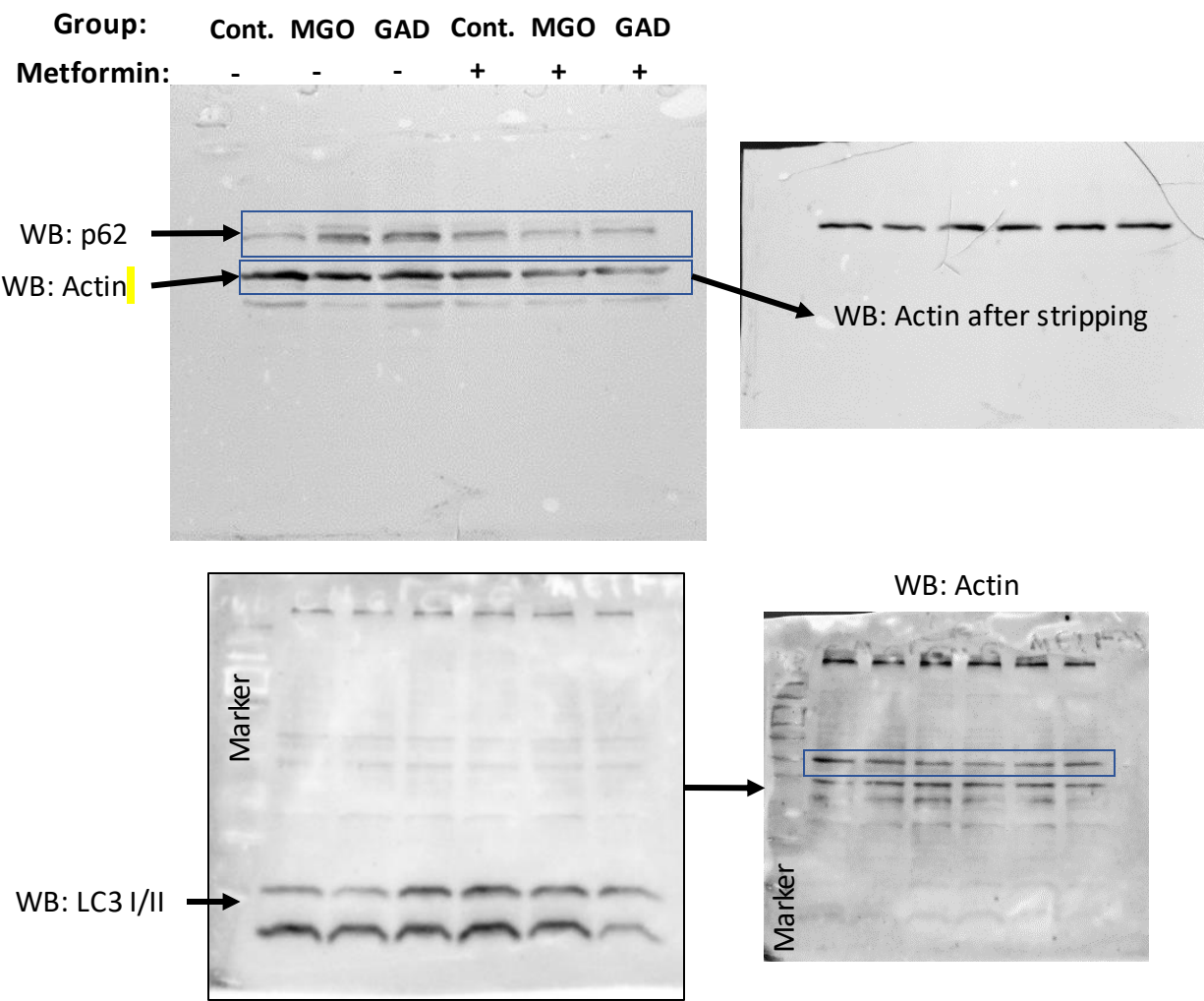

Figure 6

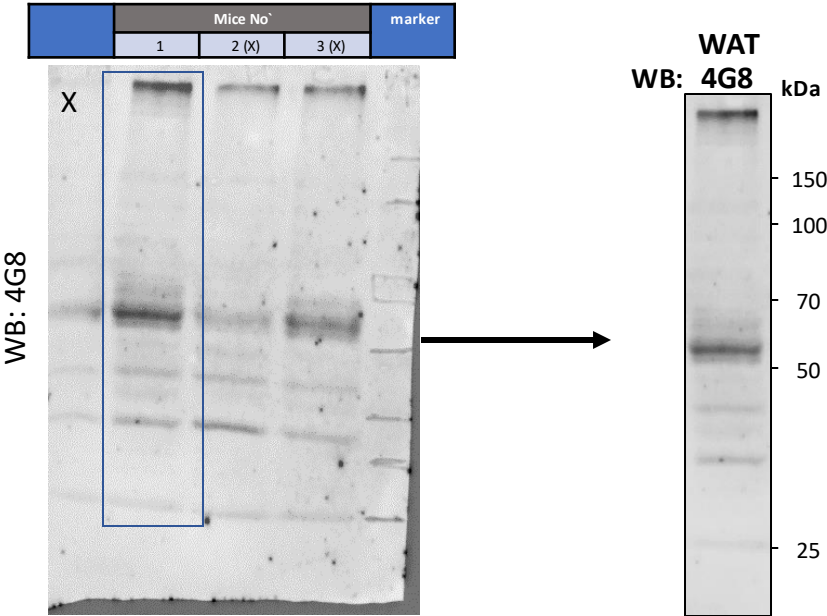

Figure 7

Figure 7- D (3T3-L1)

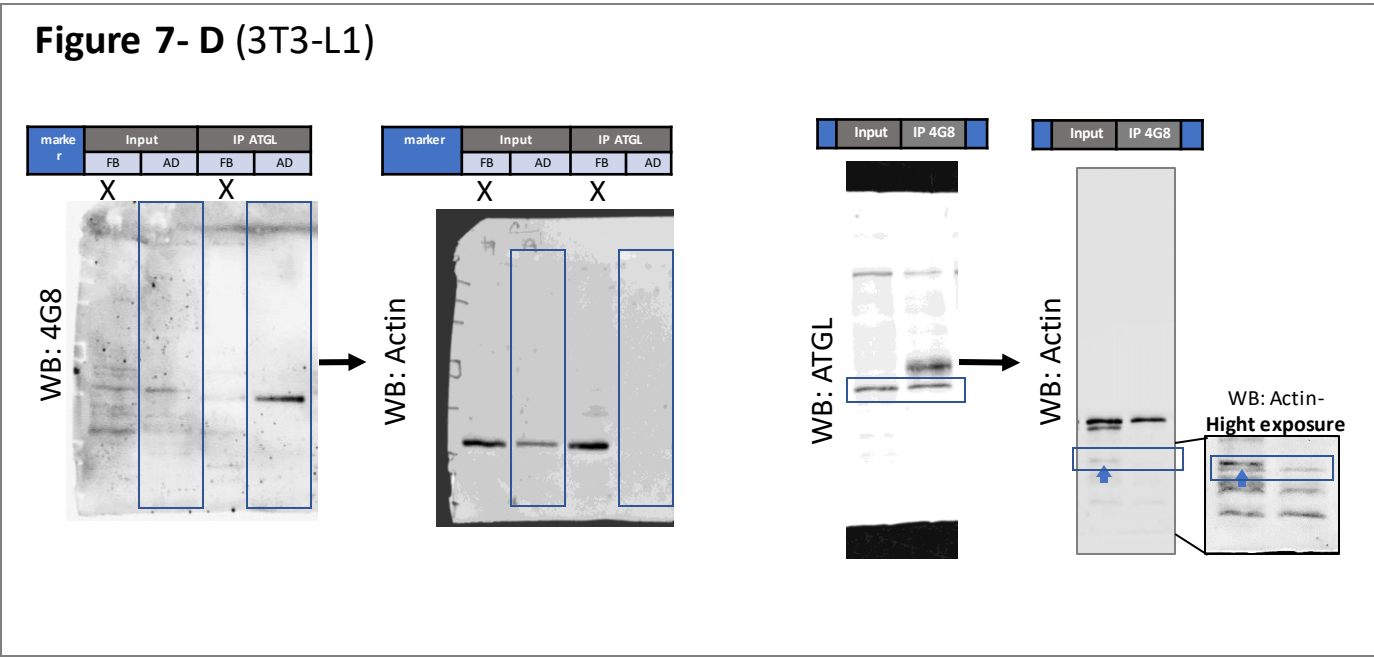

Figure 7- E (WAT)

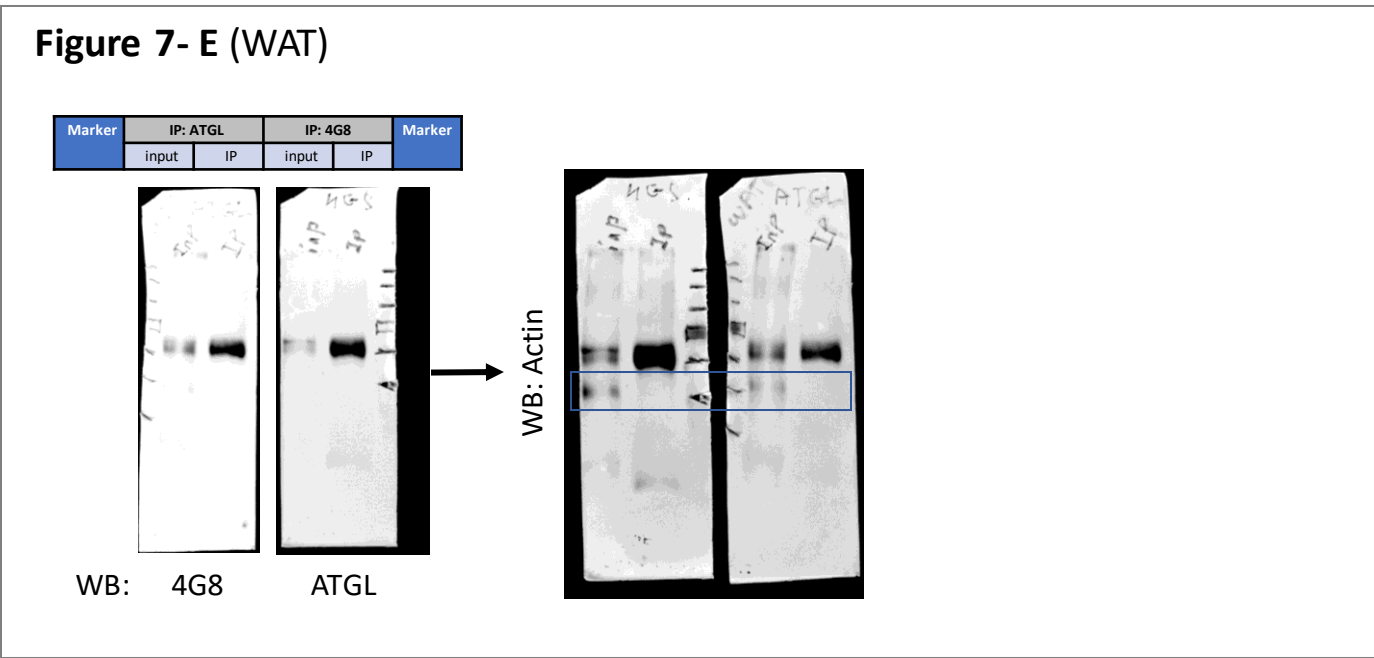

Figure S2

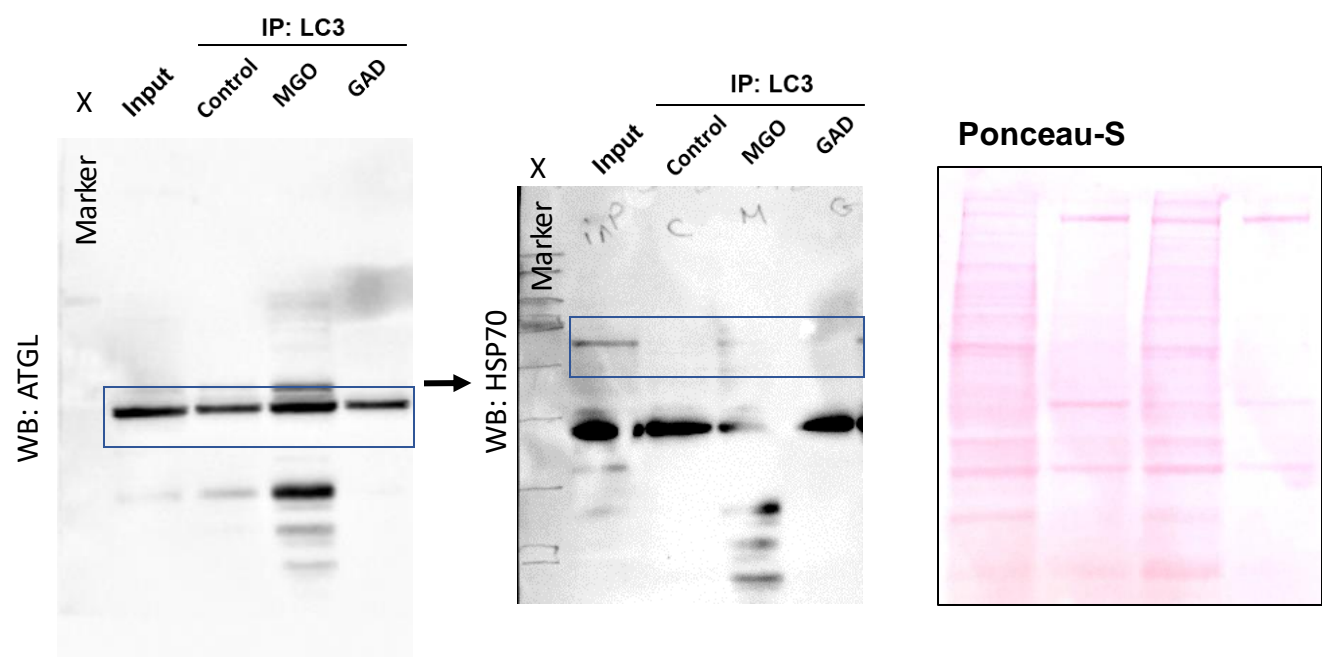

Supplement: Supplementary file 2 — Supplementary information- WB full blots [file 41419_2024_7211_MOESM2_ESM.pdf]
